# Association between Prediabetes and Renal Dysfunction from a Community-based Prospective Study

**DOI:** 10.7150/ijms.46477

**Published:** 2020-06-18

**Authors:** Chao Chen, Guangxu Liu, Xiaolan Yu, Yongbo Yu, Guangfeng Liu

**Affiliations:** 1 Xuanwu Hospital, Capital Medical University, Beijing 100053, China.; 2 National Center for Clinical Laboratories, Beijing Hospital, National Center of Gerontology; Institute of Geriatric Medicine, Chinese Academy of Medical Sciences, Beijing 100730, China.; 3 Beijing Jiangong Hospital, Beijing, 100054, China.; 4 Beijing Key Laboratory for Pediatric Diseases of Otolaryngology, Head and Neck Surgery, Beijing Children's Hospital, Capital Medical University, Beijing 100045, China.; 5 Department of Emergency, Feicheng Traditional Chinese Medicine Hospital, Taian, Shandong, 271600, China.

**Keywords:** prediabetes, renal dysfunction, association

## Abstract

**Objective:** Our study aimed to evaluate the association between prediabetes and renal dysfunction, and further assess which of glycemic indices of fasting plasma glucose (FPG), postprandial plasma glucose (PPG) and hemoglobin A1c (HbA1c) has a higher risk of renal dysfunction.

**Methods:** This was a community-based prospective cohort study, which included 7015 participants from Beijing and Taian between May and October in 2015. The outcome was the renal dysfunction defined as estimated glomerular filtration rate (eGFR)<60 mL/min/1.73 m^2^. Univariate and multivariate logistic regression model was performed, and calculated the odds ratio (OR) and 95% confidence interval (95%CI) of renal dysfunction. Receiver operating curve (ROC) analysis was used to predict renal dysfunction for glycemic indices.

**Results:** 121 renal dysfunction cases were identified. We found that the adjusted ORs (95%CI) of renal dysfunction were 1.72 (1.11-2.38), 1.48 (1.09-1.93), 1.97 (1.27-2.89) and 1.35 (1.07-2.13), respectively, for those with prediabetes, impaired fasting glucose (IFG), impaired glucose tolerance (IGT) and elevated HbA1c, compared with individuals with normal glucose tolerance. And IGT presented a higher risk of renal dysfunction than other glycemic indices. The similar results were obtained by performing the subgroup analysis. ROC analysis revealed the PPG had a higher predictive value for renal dysfunction.

**Conclusion**: We found prediabetes was positively associated with the risk of renal dysfunction and PPG had a higher risk and predictive value of renal dysfunction than other glycemic indices of FPG and HbA1c.

## Introduction

Prediabetes is considered as a condition that plasma glucose is abnormally elevated, but not up to the standard of diabetes 1. In the year of 2013, the estimated overall prevalence of prediabetes was 35.7% of Chinese adults 2. Individuals with prediabetes are at high risk of developing diabetes and complications such as kidney, eye and cardiovascular disease. Previous studies have demonstrated that diabetes could increase the risk of kidney dysfunction 3, 4, which was defined as the estimated glomerular filtration rate (eGFR) <60 mL/min/1.73 m^2^ 5. However, whether prediabetes contributes to the impairment of kidney function is not well characterized 6-10. The data based on the cross-sectional study of Cooperative Health Research in the Augsburg Region (KORA) showed that prediabetes had harmful influence on kidney function 11. On the contrary, the results from the Framingham Heart Study offspring cohort (1991-1995) found that the risk factors of cardiovascular disease, not prediabetes, may affect the development of kidney disease 10. To the best of our knowledge, it was equivocal for the association between prediabetes and kidney function among the Chinese population without diabetes 12, 13.

The plasma glucose including fasting glucose (FPG), postprandial plasma glucose (PPG) and hemoglobin A1c (HbA1c) was used to define the prediabetes according to American Diabetes Association (ADA) prediabetes diagnostic criteria. Many studies have the association between elevated plasma glucose levels and increasing risk of renal dysfunction or kidney disease. In the Atherosclerosis Risk in Communities study, the results demonstrated that HbA_1c_ were better performance of kidney disease than fasting glucose or 2-hour glucose in prediabetes population 14. However, several studies from Asian reported that elevated HbA1c or PPG, not FPG, presented an increasing risk of kidney disease 15-17. Moreover, one study from the Caucasian concluded that FPG was not associated with the development of kidney disease 18. Thus, it is discrepant for the association between glycemic indices and renal function and it is not clear that which of glycemic indices presented a close association with increasing risk of renal dysfunction.

The objective of this study was to investigate the effects of prediabetes on renal dysfunction in Chinese population without diabetes. Based on the glucose indices, we also compared the effects performance of FPG, PPG and HbA1c of prediabetes in their relationship with renal dysfunction.

## Materials and Methods

### Study participants

**Figure 1:**
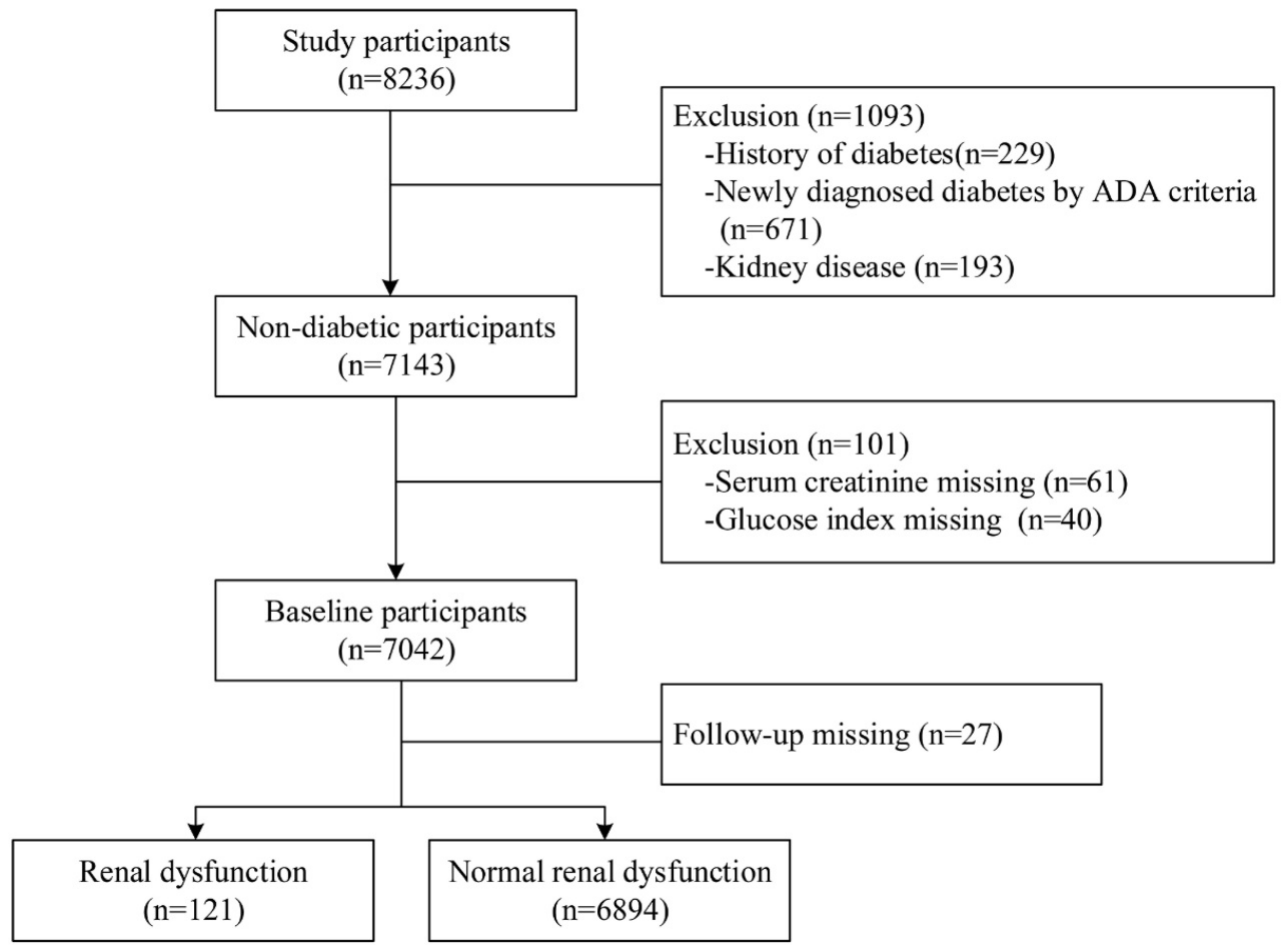
Flowchart of the study population and the incident of renal dysfunction during follow-up time.

The participants were derived from a community-based prospective cohort study in Shijingshan district of Beijing and Taian city of Shandong Province. The participants who were aged 40 years and older and identified by the local residence registration systems were included and there was no restriction on gender or ethnicity. From May to October of 2015, 8236 participants were invited by the trained community workers and conducted the follow-up by face-to-face interview during July and December, 2018. Among them, we excluded 1093 participants who had already kidney disease and diabetes at baseline and 101 participants who had missing key variables. We further excluded 27 participants with lost to follow-up, and thus there were 7015 participants in this analysis (Figure 1). All of those participants gave written informed consent in accordance with the Declaration of Helsinki. The investigation was approved by the Committee on Human Research of the Xuanwu Hospital, Capital Medical University.

### Data Collection

At baseline, in order to collect information on demographics, history of disease and corresponding medication use, smoking status and drinking status, all participants were asked to complete a standard questionnaire by a face-to-face interview. Information on weight, height and blood pressure was obtained through physical examination conducted by trained personnel. Weight was measured to the nearest 0.5 kg by an electronic weight scale with participants wearing only light clothing and after emptying the bladder. Height was measured with the participants removing their shoes, overcoats, and empties their pockets, using a vertical height meter to the nearest 0.1 cm. Body mass index (BMI) was calculated as weight (kilograms) divided by height (meters) squared (kg/m^2^). Blood pressure was measured in triplicate after a 5-min rest, using a mercury sphygmomanometer, and the mean value of the three readings was recorded. All participants provided 10 mL of blood sample after an overnight fast (at least 10 h) for biochemical analyses, including HbA1c, total cholesterol (TC), triglycerides (TG), high density lipoprotein cholesterol (HDL-C), low density lipoprotein cholesterol (LDL-C) and serum creatinine (Scr). The Scr assay was measured with an autoanalyzer (c16000 system, ARCHITECT ci16200 analyzer; Abbott Laboratories, Chicago, IL, USA), which was standardized to an isotope dilution mass spectrometry reference measurement procedure. Estimated glomerular filtration rate (eGFR), which expressed in mL/min per 1.73 m^2^, was calculated on the basis of Scr, using the Chronic Kidney Disease Epidemiology Collaboration (kidney disease-EPI) formula: Male: if Scr ≤ 0.9 mg/dL then eGFR= 141 × (Scr /0.9) ^- 0.411^ × (0.993) ^age^, if Scr > 0.9 mg/dL: eGFR= 141 × (Scr/ 0.9) ^- 1.209^ × (0.993) ^age^. Female: if Scr ≤ 0.7 mg/dL then eGFR= 144 × (Scr /0.7) ^- 0.329^ × (0.993) ^age^, if Scr > 0.7 mg/dL then eGFR= 144 × (Scr /0.7) ^- 1.209^ × (0:993) ^age^.

### Definitions of key variates

Renal dysfunction was defined as eGFR < 60 mL/min per 1.73 m^2^. Prediabetes was defined as fasting plasma glucose (FPG) 5.6-6.9 mmol/L (100-125 mg/dl), or postprandial plasma glucose (PPG) 7.8-11.0 mmol/l (140-199 mg/dl) or HbA1c 5.7-6.5% (39-47mmol/mol) according to the ADA criteria^19^. Impaired fasting glucose (IFG) was defined as FPG ≥ 5.6 mmol/L and < 7.0 mmol/L. Impaired glucose tolerance (IGT) was defined as PPG ≥ 7.8 mmol/L and < 11.0 mmol/L. Elevated HbA1c (EHbA1c) was defined as HbA1c ≥5.7% and < 6.5%. Hypertension was defined as systolic blood pressure (SBP) ≥140mmHg and /or diastolic blood pressure (DBP) ≥90mmHg from the mean of three measurements taken at baseline or being previously diagnosed by clinicians.

### Statistical Analysis

For continuous variables, data was expressed as means ± standard deviations (SD), and data was expressed as frequency and percentages for categorical variables. We used t test, Kruskal-Wallis test or chi-square test to compare the baseline characteristics between normal glucose tolerance (NGT) and prediabetes. Logistic regression models were used to estimate the association between prediabetes and incident of kidney disease, by three step forward multivariable-adjusted models: model 1 unadjusted; model 2 adjusted for age, sex, BMI; model 3 adjusted for age, sex, BMI, TC, TG, HDL-C, LDL-C, SBP, and DBP. For further analyses, we conducted subgroup analysis stratified by sex and hypertension status, which adjusted all potential confounding factors. We further used the receiver-operating characteristic (ROC) curve analysis to assess the prediction of glycemic indices for events of renal dysfunction and calculate the Youden index for the occurrence of renal dysfunction, according to the ADA and WHO criteria. All statistical analyses were performed with the Statistical Analysis System (SAS) version 9.4 (SAS Institute Inc., Cary, NC, USA). Forest plots were drawn using the Graphpad Prism 7 (Graphpad Software Company, CA, USA). All tests were two-sided, and a value of P < 0.05 was considered as significant.

## Results

### Characteristics of participants at baseline

Among 8326 participants at baseline, we excluded 1093 participants with diabetes, 101 participants with key variable missing and 27 participants loss to follow-up. Finally, a total of 7015 participants were included in this study (Figure 1). The mean age of the study population was 57.3±7.4 years, and 4704 (67.1%) were female. According to the ADA criteria, 4321 participants had prediabetes and 2694 participants had NGT. Compared to the participants of NGT, the prediabetes participants were older and had higher levels of BMI, SBP, TC, TG, LDL-C, and lower levels of DBP, HDL-C (Table 1).

### Association prediabetes defined by ADA criteria with renal dysfunction

There were 121 (1.7%) cases of renal dysfunction incidents during follow-up period. The odds ratio (OR) (95%CI) of renal dysfunction in participants with prediabetes, was 1.91 (1.46-2.96), respectively, compare with the participants with NGT. And the ORs (95%CI) of renal dysfunction in the phenotypes categorized by glycemic indices were 1.67 (1.34-2.72) with impaired fasting glucose (IFG), 2.38 (1.72-3.86) with IGT and 1.56 (1.26-2.37) with EHbA1c than the NGT group. After adjustment for age, sex, BMI, TC, TG, HDL-C, LDL-C, SBP, and DBP, the prediabetes group still presented a significantly higher occurrence risk of renal dysfunction than NGT (OR=1.72, 95%CI: 1.11-2.38). Furthermore, the similar results were shown in group of IFG, IGT and EHbA1c, but the IGT group had a higher risk of renal dysfunction (OR=1.97, 95%CI: 1.27-2.89) than the group of IFG and EHbA1c, after adjusting for all confounding factors (Table 2).

### Subgroup analysis of the association between prediabetes and renal dysfunction

**Figure 2:**
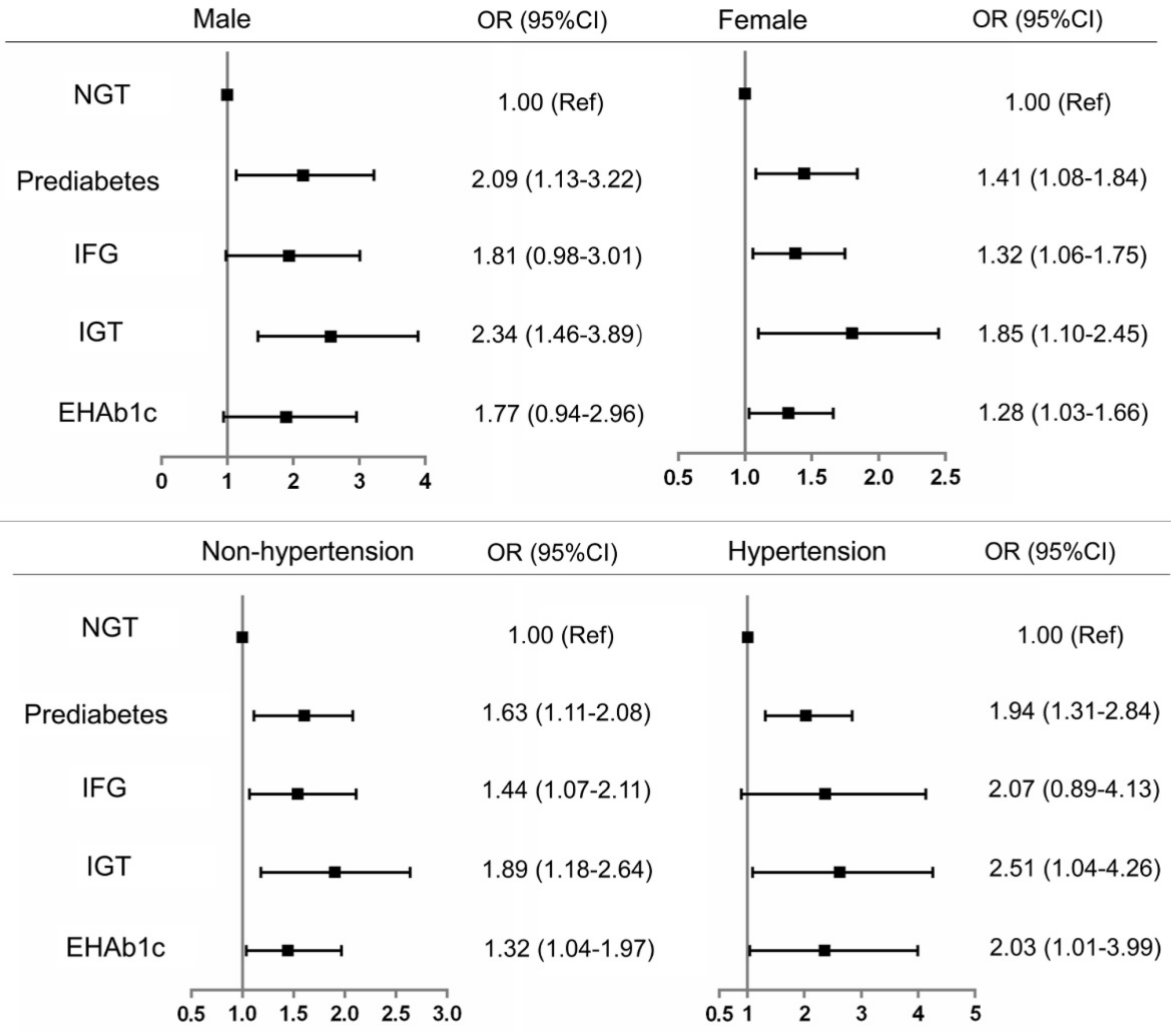
Subgroup analysis of risk of renal dysfunction with prediabetes defined by ADA criteria NGT: normal glucose tolerance HbA1c: glycosylated hemoglobin. IFG: impaired fasting glucose. IGT: impaired glucose tolerance. EHbA1c: Elevated glycated hemoglobin A1c. OR: odds ratio. CI: confidence interval.

To further confirm the association of renal dysfunction with prediabetes and its phenotypes, we conducted subgroup analysis by sex and hypertension. The results showed that prediabetes and its phenotypes had a higher risk of renal dysfunction than the NGT participants after simultaneous adjustment for all potential confounding factors. However, only the phenotype of IGT was significantly associated with the risk of renal dysfunction (OR=2.34, 95%CI: 1.46-3.89) among male participants. Whereas, all participants with prediabetes, IFG, IGT and EHbA1c presented a higher risk of renal dysfunction than NGT participants, whether hypertension or not (Figure 2).

### Predictive value of glycemic indices for renal dysfunction

ROC curve analysis was used to compare which glycemic indices of FPG, PPG and HbA1c could predict the events of renal dysfunction. The data showed that the area under curve (AUC) of PPG (AUC=0.63, 95%CI: 0.61-0.65) was higher than FPG (AUC=0.58, 95%CI: 0.56-0.61) and HbA1c (AUC=0.60, 95%CI: 0.58-0.61).The optimal cut-off values of predicting the occurrence of renal dysfunction were FPG ≥5.57 mmol/L, PPG ≥7.29 mmol/L and HbA1c ≥5.65%, which were mostly similar to those the cut-off values based on ADA criteria but were higher than those cut-off points of glycemic indices of FPG and PPG based on WHO criteria. Moreover, PPG had a higher Youden index than other glycemic indices in predicting renal dysfunction incidents.

## Discussion

In this study, we found the prediabetes was positively associated with the renal dysfunction in Chinese middle aged and elderly population. The phenotype of prediabetes defined by glycemic indices indicated that the participants with IFG, IGT and EHbA1c all increased the risk of renal dysfunction compared with NGT participants, and IGT exhibited a higher risk of renal dysfunction. Furthermore, PPG presented a superior predictive effect for renal dysfunction.

Evidences showed that prediabetes was positive associated with renal function. Two prospective cohort studies revealed that prediabetes increased 40% 20 and 80% 7 risk of kidney disease, respectively. A meta-analysis including nine published cross-sectional or cohort studies completed before 2015 also indicated that prediabetes significantly increased 11% occurrence of renal function or kidney disease (OR=1.11, 95%CI: 1.02-1.21) 21. Recently, a prospective cohort study including 7728 Korean adults showed that prediabetes was an independent risk factor for renal dysfunction 17. And our findings also confirmed the positive association between prediabetes and renal dysfunction. On the contrary, a cross-sectional study 22 and another cohort study 8 indicated that prediabetes was not a risk factor for renal dysfunction. The inconsistence of these results could be explained by the discrepancies of definition of prediabetes using only IFG or elevated HbA1c, not IGT. Therefore, some participants with impaired postprandial plasma glucose maybe misclassify to “NGT” individuals. In our study, the definition of prediabetes using three glycemic indices of FPG, PPG and HbA1c made these result reliable and convincing. In our study, we found PPG had a higher risk of renal dysfunction. The reason might be that the postprandial plasma glucose is characterized by the rapid and large increase of blood glucose concentrations, which produces an increase of glomerular filtration rate in kidney 23. The hyperperfusion results in a greater stimulus for hyperproduction of collagen which was considered an important event for pathogenesis of diabetic nephropathy 24. Simultaneously, blood flow was also closely parallel with plasma glucose concentrations 25, which both pathogenetic factors are important to the occurrence and progression of kidney dysfunction.

There are some major strengths in this study. It was a multi-community and well-designed prospective study, which confirmed a robust and convincing conclusion. Furthermore, simultaneous assessment of glycemic indices of FPG, PPG and HbA1c could identify as many prediabetes participants as possible, thus avoiding selective bias caused by misdiagnosis. Lastly, this study benefited from detailed information of standardized procedures such as face-to-face visit, physical examination and laboratory test for each participant, guaranteeing to collect all potential confounding factors. However, there are some limitations in this study. Firstly, participants enrolled were aged 40 years and more, which may produce potential bias and poor extrapolation. Secondly, urinary albumin was not detected in study investigation, which leads to underestimate of renal dysfunction participant. Thirdly, the relatively short follow-up time resulted in few renal dysfunction events, and further study of delaying follow-up time will be necessary for the reproducibility.

In summary, our findings showed prediabetes was positively associated with renal dysfunction in Chinese middle-aged and elderly. PPG presented a higher risk and stronger predictive value for renal dysfunction. The results emphasize that prediabetes should be pay more attention, especially for IGT individuals, in order to attenuate the risk of renal dysfunction even nephropathy occurrence.

## Figures and Tables

**Table 1:** Baseline characteristics of study participants

Characteristics	NGT (n=2694)	Prediabetes (n=4321)	P value
Age (years)	56.0±7.1	58.2±7.5	<0.001
Female, n (%)	1744 (64.7)	2960 (68.5)	<0.001
Current smoking, n (%)	523 (19.4)	1288 (29.8)	<0.001
Current drinking, n (%)	816 (30.3)	1577 (36.5)	<0.001
Physical exercise, n (%)	1018 (37.8)	1400 (32.4)	<0.001
BMI (kg/m^2^)	24.9±3.2	25.9±3.4	<0.001
HbA1c (%)	5.4 (5.3-5.5)	5.9 (5.8-6.1)	<0.001
SBP (mmHg), n (%)	128.5±16.7	132.2±16.7	<0.001
DBP (mmHg), n (%)	76.2±14.0	75.4±10.6	0.020
Hypertension, n (%)	633 (23.5)	1286 (29.8)	<0.001
TC (mmol/L)	5.0±0.6	5.3±0.6	<0.001
TG (mmol/L)	1.2±0.4	1.5±0.3	<0.001
HDL-C (mmol/L)	1.4±0.2	1.3±0.3	0.034
LDL-C (mmol/L)	3.0±0.4	3.3±0.5	<0.001
eGFR (mL/min/1.73 m2), Median (IQR)	96.1 (87.3-102.3)	93.5 (86.4-101.6)	0.001

Data were expressed as mean ± standard deviation or frequency (%), unless specified; P value: from t test, Kruskal-Wallis test or chi-square test; Abbreviations: NGT: Normal glucose tolerance; BMI: Body mass index; HbA1c: Glycosylated hemoglobin; SBP: Systolic blood pressure; DBP: Diastolic blood pressure; TC: Total cholesterol; TG: Triglycerides; HDL-C: Fasting serum high density lipoprotein cholesterol; LDL-C: Low density lipoprotein cholesterol; eGFR: Estimated glomerular filtration rate. IQR: Interquartile range.

**Table 2:** Risk of renal dysfunction with prediabetes defined by ADA criteria

	Events/N			Model 2		Model 3	
		OR (95%CI)	P value	OR (95%CI)	P value	OR (95%CI)	P value
NGT	30/2694 (1.11)	1.00		1.00		1.00	
Prediabetes	91/4321 (2.10)	1.91 (1.46-2.96)	<0.001	1.83(1.23-2.79)	0.001	1.72(1.11-2.38)	0.001
IFG	34/1831(1.84)	1.67 (1.34-2.72)	<0.001	1.52(1.21-2.08)	0.004	1.48(1.09-1.93)	0.004
IGT	42/1589(2.64)	2.38 (1.72-3.86)	<0.001	2.12(1.41-3.03)	<0.001	1.97(1.27-2.89)	<0.001
EHbA1c	62/3607(1.71)	1.56 (1.26-2.37)	<0.001	1.40 (1.22-2.40)	0.003	1.35(1.07-2.13)	0.008

OR and 95%CI are from logistic regression; Model 1 unadjusted for covariates; Model 2 adjusted for age, sex and BMI; Model 3 adjusted for age, sex, BMI, TC, TG, HDL-C, LDL-C, SBP, and DBP. NGT: normal glucose tolerance HbA1c: glycosylated hemoglobin. IFG: impaired fasting glucose. IGT: impaired glucose tolerance. EHbA1c: Elevated glycated hemoglobin A1c. OR: odds ratio. CI: confidence interval.

**Table 3:** Receiver operating curve analysis of glucose indices for prediction of renal dysfunction

	AUC (95%CI)	Sensitivity (%)	Specificity (%)	Youden index	Cutoff point
ROC					
FPG	0.58 (0.56-0.61)	54.6 (45.2-63.1)	66.1 (57.4-75.6)	0.207	5.57 mmol/L
PPG	0.63 (0.61-0.65)	68.3 (59.5-76.4)	66.7 (58.5-75.1)	0.350	7.29 mmol/L
HbA1c	0.60 (0.58-0.61)	71.3 (61.1-80.6)	55.4 (44.7-64.2)	0.267	5.65 %
**ADA criteria**					
FPG	-	54.4 (45.3-64.1)	65.5 (56.3-74.9)	0.199	5.6 mmol/L
PPG	-	50.7 (39.8-59.9)	71.4 (62.0-80.2)	0.221	7.8 mmol/L
HbA1c	-	75.8 (65.5-84.7)	51.3 (40.4-61.4)	0.271	5.7 %
**WHO criteria**					
FPG	-	32.1 (21.1-43.5)	86.2 (81.3-90.4)	0.183	6.1 mmol/L
PPG	-	50.7 (39.5-59.8)	71.4 (66.2-76.7)	0.221	7.8 mmol/L

AUC: area under curve. FPG: fasting plasma glucose. PPG: postprandial plasma glucose. ADA: American Diabetes Association.

## Abbreviations

ADA: America diabetes association
AUC: area under curve
CI: confidence interval
EHbA1c: elevated hemoglobin A1c
FPG: fasting plasma glucose
HDL-C: high density lipoprotein cholesterol
IFG: impaired fasting glucose
IGT: impaired glucose tolerance
LDL-C: low density lipoprotein cholesterol
OR: odds ratio
PPG: postprandial plasma glucose
ROC: receiver operating characteristic curve
TC: total cholesterol
TG: triglyceride
WHO: world health organization

## References

[1] Canadian Diabetes Association Clinical Practice Guidelines Expert C, Goldenberg R, Punthakee Z (2013). Definition, classification and diagnosis of diabetes, prediabetes and metabolic syndrome. Can J Diabetes.

[2] Wang L, Gao P, Zhang M, Huang Z, Zhang D, Deng Q, Li Y, Zhao Z, Qin X, Jin D (2017). Prevalence and Ethnic Pattern of Diabetes and Prediabetes in China in 2013. Jama.

[3] Selvin E, Ning Y, Steffes MW, Bash LD, Klein R, Wong TY, Astor BC, Sharrett AR, Brancati FL, Coresh J (2011). Glycated hemoglobin and the risk of kidney disease and retinopathy in adults with and without diabetes. Diabetes.

[4] Jadhakhan F, Marshall T, Ryan R, Gill P (2018). Risk of chronic kidney disease in young adults with impaired glucose tolerance/impaired fasting glucose: a retrospective cohort study using electronic primary care records. BMC nephrology.

[5] Drawz P, Rahman M (2015). Chronic kidney disease. Annals of internal medicine.

[6] Ali MK, Bullard KM, Saydah S, Imperatore G, Gregg EW (2018). Cardiovascular and renal burdens of prediabetes in the USA: analysis of data from serial cross-sectional surveys, 1988-2014. The lancet Diabetes & endocrinology.

[7] Watanabe H, Obata H, Watanabe T, Sasaki S, Nagai K, Aizawa Y (2010). Metabolic syndrome and risk of development of chronic kidney disease: the Niigata preventive medicine study. Diabetes/metabolism research and reviews.

[8] Schottker B, Brenner H, Koenig W, Muller H, Rothenbacher D (2013). Prognostic association of HbA1c and fasting plasma glucose with reduced kidney function in subjects with and without diabetes mellitus. Results from a population-based cohort study from Germany. Preventive medicine.

[9] Tozawa M, Iseki C, Tokashiki K, Chinen S, Kohagura K, Kinjo K, Takishita S, Iseki K (2007). Metabolic syndrome and risk of developing chronic kidney disease in Japanese adults. Hypertension research: official journal of the Japanese Society of Hypertension.

[10] Fox CS, Larson MG, Leip EP, Meigs JB, Wilson PW, Levy D (2005). Glycemic status and development of kidney disease: the Framingham Heart Study. Diabetes care.

[11] Markus MRP, Ittermann T, Baumeister SE, Huth C, Thorand B, Herder C, Roden M, Siewert-Markus U, Rathmann W, Koenig W (2018). Prediabetes is associated with microalbuminuria, reduced kidney function and chronic kidney disease in the general population: The KORA (Cooperative Health Research in the Augsburg Region) F4-Study. Nutrition, metabolism, and cardiovascular diseases: NMCD.

[12] Zhou Y, Echouffo-Tcheugui JB, Gu JJ, Ruan XN, Zhao GM, Xu WH, Yang LM, Zhang H, Qiu H, Narayan KM (2013). Prevalence of chronic kidney disease across levels of glycemia among adults in Pudong New Area, Shanghai, China. BMC nephrology.

[13] Sun F, Tao Q, Zhan S (2010). Metabolic syndrome and the development of chronic kidney disease among 118 924 non-diabetic Taiwanese in a retrospective cohort. Nephrology (Carlton, Vic).

[14] Warren B, Pankow JS, Matsushita K, Punjabi NM, Daya NR, Grams M, Woodward M, Selvin E (2017). Comparative prognostic performance of definitions of prediabetes: a prospective cohort analysis of the Atherosclerosis Risk in Communities (ARIC) study. The lancet Diabetes & endocrinology.

[15] Wang C, Song J, Sun Y, Hou X, Chen L (2014). Blood glucose is associated with chronic kidney disease in subjects with impaired glucose tolerance, but not in those with impaired fasting glucose. Journal of diabetes.

[16] Koshi T, Sagesaka H, Sato Y, Hirabayashi K, Koike H, Yamauchi K, Nishimura R, Noda M, Yamashita K, Aizawa T (2018). Elevated haemoglobin A1c but not fasting plasma glucose conveys risk of chronic kidney disease in non-diabetic individuals. Diabetes research and clinical practice.

[17] Kim GS, Oh HH, Kim SH, Kim BO, Byun YS (2019). Association between prediabetes (defined by HbA1C, fasting plasma glucose, and impaired glucose tolerance) and the development of chronic kidney disease: a 9-year prospective cohort study. BMC nephrology.

[18] Vieira MB, Neves JS, Leitao L, Baptista RB, Magrico R, Dias CV, Oliveira A, Carvalho D, Mc Causland FR (2019). Impaired Fasting Glucose and Chronic Kidney Disease, Albuminuria, or Worsening Kidney Function: a Secondary Analysis of the SPRINT. The Journal of clinical endocrinology and metabolism.

[19] [Book] Classification and Diagnosis of Diabetes (2018). Standards of Medical Care in Diabetes-2018. Diabetes care.

[20] Lucove J, Vupputuri S, Heiss G, North K, Russell M (2008). Metabolic syndrome and the development of CKD in American Indians: the Strong Heart Study. American journal of kidney diseases: the official journal of the National Kidney Foundation.

[21] Echouffo-Tcheugui JB, Narayan KM, Weisman D, Golden SH, Jaar BG (2016). Association between prediabetes and risk of chronic kidney disease: a systematic review and meta-analysis. Diabetic medicine: a journal of the British Diabetic Association.

[22] Xing FY, Neeland IJ, Gore MO, Ayers CR, Paixao AR, Turer AT, Berry JD, Khera A, de Lemos JA, McGuire DK (2014). Association of prediabetes by fasting glucose and/or haemoglobin A1c levels with subclinical atherosclerosis and impaired renal function: observations from the Dallas Heart Study. Diabetes & vascular disease research.

[23] Skott P, Vaag A, Hother-Nielsen O, Andersen P, Bruun NE, Giese J, Beck-Nielsen H, Parving HH (1991). Effects of hyperglycaemia on kidney function, atrial natriuretic factor and plasma renin in patients with insulin-dependent diabetes mellitus. Scandinavian journal of clinical and laboratory investigation.

[24] Steffes MW, Bilous RW, Sutherland DE, Mauer SM (1992). Cell and matrix components of the glomerular mesangium in type I diabetes. Diabetes.

[25] Monnier L, Lapinski H, Colette C (2003). Contributions of fasting and postprandial plasma glucose increments to the overall diurnal hyperglycemia of type 2 diabetic patients: variations with increasing levels of HbA(1c). Diabetes care.

